# Full-Length Transcriptome Comparison of Male and Female Gonads in *Scatophagus argus* Reveals Alternative Splicing Events

**DOI:** 10.3390/biom16091270

**Published:** 2026-09-02

**Authors:** Fangyuan Qin, Kaizhi Jiao, Fei Zhi, Yu Li, Siping Deng, Tianli Wu, Dongneng Jiang

**Affiliations:** Guangdong Research Center on Reproductive Control and Breeding Technology of Indigenous Valuable Fish Species, Guangdong Provincial Key Laboratory of Aquatic Animal Disease Control and Healthy Culture, Fisheries College, Guangdong Ocean University, Zhanjiang 524088, China; qinfy@stu.gdou.edu.cn (F.Q.); jiaokaizhi732@gmail.com (K.J.);

**Keywords:** *Scatophagus argus*, alternative splicing, full-length transcript, gametogenesis

## Abstract

Spotted scat (*Scatophagus argus*) is an economically important fish species with an XX/XY sex determination (SD) system and the candidate sex-determining gene *Dmrt1Y*. Alternative splicing (AS) contributes to transcript diversity and post-transcriptional regulation, but its role in fish gonadal function remains unclear. In this study, we conducted a comprehensive analysis of AS events in adult ovaries (*n* = 3) and testes (*n* = 3) of *S. argus* using ONT-based full-length transcriptome sequencing. An average of 7.30 Gb of clean data per sample was obtained, with a mean N50 length of 1502 bp. A total of 3333 differentially expressed genes (DEGs) were identified, including 2064 ovary-upregulated and 1269 testis-upregulated genes. Additionally, 9417 and 17,216 AS events were detected in the ovary and testis, respectively. Notably, no AS events were detected in the *Dmrt1Y* gene. A total of 2253 differential alternative splicing (DAS) events involving 1300 genes were identified between ovaries and testes. Of these, only 213 genes were also differentially expressed. Sex-biased AS patterns were observed in genes potentially associated with gonadal function and germ-cell regulation, including *Ncoa5*, *Tp53*, and *Fancl*. Male-biased AS events in *Tp53* and *Fancl* were predicted to alter coding regions corresponding to the DNA-binding and UBC-like domains, respectively. Furthermore, male-biased *Ncoa5* isoforms lacking exons 3 and 4 (381 bp) were predicted to result in partial loss of the RNA recognition motif (RRM). Collectively, this study provides insights into AS regulation in adult gonads of *S. argus* and highlights the potential contribution of AS to reproduction.

## 1. Introduction

Studies on gonadal development in fish have garnered significant interest due to their potential applications in aquaculture [1]. However, the role of alternative splicing (AS) in regulating gene expression and contributing to gonadal development remains underexplored, particularly in fish. AS is a widespread regulatory mechanism that generates multiple mRNA isoforms from a single gene through differential exon or intron usage, thereby increasing proteomic diversity [2,3]. This process can alter open reading frames (ORFs) or untranslated regions (UTRs), influencing mRNA stability, localization, and translation, ultimately producing protein isoforms with distinct functions or subcellular localizations [4,5]. In humans, up to 95% of genes undergo AS, with many producing multiple protein isoforms [6,7]. Notably, sex-specific AS events, in which distinct exons are differentially spliced between males and females, have been implicated in vertebrate gonadal development [8,9]. Although studies in model organisms such as *Drosophila* and mice have demonstrated that sex-specific splicing contributes to gonadal development [10,11], its contribution to these processes in teleosts remains poorly understood. AS events in sex-related genes have also been associated with gonadal development-related processes in teleosts, such as *Dmrt1* in the rice field eel (*Monopterus albus*) and barramundi (*Lates calcarifer*) [12,13]. However, genome-wide identification of AS events in teleost gonads has not been systematically conducted, and the regulatory networks linking AS to sex-biased gene expression remain largely unknown.

*Scatophagus argus* is an economically important marine fish species with a genetically stable XX/XY sex determination system. In recent years, the availability of extensive genomic resources has provided a solid foundation for investigating the molecular mechanisms underlying gonadal development and differentiation in this species [14,15]. Female *S. argus* exhibit higher growth rates than males [16], making all-female populations highly desirable for aquaculture. However, attempts to generate XX neo-males through sex reversal have achieved limited success, which may be attributed to the absence of a functional *Dmrt1* gene in genetic females [17,18]. Therefore, a deeper understanding of the molecular networks underlying gonadal development is essential to enhance sex-control breeding strategies. Although previous transcriptome studies using short-read NGS technology have identified numerous sex-biased genes associated with gonadal development in *S. argus* [19], this technology has limited capacity for accurately reconstructing full-length transcripts and resolving complex alternative splicing (AS) events.

To investigate the potential roles of AS events in adult gonads of *S. argus*, this study utilized third-generation Oxford Nanopore Technologies (ONT) sequencing to generate full-length transcriptome data from stage IV ovaries and testes (three biological replicates per sex). By overcoming the limitations of short-read sequencing, we systematically analyzed global AS events, differentially expressed genes (DEGs), and differentially spliced genes (DSGs) in the gonads of *S. argus*. These findings provide a comprehensive resource for understanding AS-mediated post-transcriptional regulation in adult gonadal tissues and offer new insights into the potential roles of AS in gonadal development in *S. argus*.

## 2. Materials and Methods

### 2.1. Animals and Sample Collection

Six adult *S. argus* (three females and three males) were collected from the Donghai Island Breeding Base in Zhanjiang, Guangdong Province, China. Before sampling, the fish were anesthetized with 10 mg/L 4-allyl-2-methoxyphenol (E809010, Macklin, Shanghai, China). After anesthesia, a rapid dissection was performed to remove the gonads. One portion of each gonad was immediately frozen in liquid nitrogen and stored at −80 °C until RNA extraction. The remaining portion was fixed in 4% paraformaldehyde (Sigma-Aldrich, St. Louis, MO, USA) for histological examination. Histological characterization confirmed that all gonads were at stage IV, as previously reported by Jiao et al. (2024) [20]. No endangered or protected species were used in this study. All animal experiments were conducted in accordance with the Animal Research and Ethics Committee of Guangdong Ocean University (No. 201903004).

### 2.2. Total RNA Extraction, Library Construction, and Full-Length RNA Sequencing

Total RNA was extracted using TRIzol reagent (Invitrogen, Carlsbad, CA, USA) according to the manufacturer’s instructions. RNA concentration and purity were measured using a NanoDrop 2000 spectrophotometer (Thermo Fisher Scientific, Waltham, MA, USA), while RNA integrity was evaluated by 1.5% agarose gel electrophoresis and an Agilent 2100 Bioanalyzer (Agilent Technologies, Santa Clara, CA, USA). Only high-quality RNA samples were used for subsequent library construction.

Full-length cDNA libraries were constructed by Biomarker Technologies Corporation (Beijing, China) according to the manufacturer’s instructions. Briefly, poly(A) mRNA was enriched from 1 μg of total RNA using mRNA capture beads (Vazyme, Nanjing, China). Reverse transcription was then performed using the SuperScript IV First-Strand Synthesis System (Invitrogen) with customized primers, followed by cDNA PCR amplification for 14 cycles with LongAmp Taq (New England Biolabs, Ipswich, MA, USA). The resulting double-stranded DNA products were subjected to damage repair and end-preparation using the NEBNext FFPE DNA Repair Mix and the NEBNext Ultra II End Repair/dA-Tailing Module (NEB). Sequencing adapters were subsequently ligated using the Ligation Sequencing Kit (SQK-LSK109, Oxford Nanopore Technologies, Oxford, UK). After purification and quality assessment, the libraries were loaded onto PromethION Flow Cells (FLO-PRO002, ONT) and sequenced on a PromethION 48 platform according to the manufacturer’s instructions. The raw sequencing data have been deposited in the NCBI Sequence Read Archive (SRA) under accession number PRJNA937450.

### 2.3. RNA-Seq Data Processing and Differential Expression Analysis

Raw ONT reads were first filtered to remove low-quality reads with an average quality score < 7 and read length < 500 bp. Ribosomal RNA sequences were removed by mapping reads to an rRNA database. Full-length non-chimeric (FLNC) transcripts were identified by detecting primers at both ends of the reads. FLNC reads were mapped to the reference genomes [14] using Minimap2 v2.24 with splice-aware parameters. Gene-level expression was quantified as raw read counts by assigning mapped FLNC reads to annotated genes. Differential expression analysis between ovary and testis samples was performed using the DESeq2 R package (v1.30.1). DESeq2 uses raw counts as input and estimates size factors and dispersion parameters based on a negative binomial model. *p*-values were adjusted for multiple testing using the Benjamini–Hochberg method to control the false discovery rate (FDR). Genes with |log2 fold change| > 2.0 and FDR < 0.05 were considered significantly differentially expressed.

### 2.4. AS Analysis and Differential Alternative Splicing (DAS) Identification

The transcript isoforms identified from the ONT full-length transcriptome were used as input for SUPPA2 (v2.3) [21] to identify and quantify alternative splicing (AS) events. Seven types of AS events were analyzed, including exon skipping (SE), mutually exclusive exons (MX), retained introns (RI), alternative first exon (AF), alternative last exon (AL), alternative 5′ splice site (A5), and alternative 3′ splice site (A3). For each AS event, SUPPA2 calculated the Percent Spliced-In (PSI) values for each sample and estimated differential PSI (dPSI) between ovaries and testes. The differential alternative splicing (DAS) events were screened based on an absolute |dPSI| > 0.1 and an FDR-adjusted *p*-value < 0.05. Multiple-testing correction was performed using the Benjamini–Hochberg method implemented in SUPPA2. Genes containing DAS events are referred to as differential AS genes (DSGs). To ascertain their biological roles, Gene Ontology (GO) enrichment analysis was conducted using the Metascape online platform (https://metascape.org). Furthermore, Kyoto Encyclopedia of Genes and Genomes (KEGG) enrichment analysis was performed based on the KEGG annotation results of *S. argus* genes (https://www.genome.jp/kegg/ (accessed on 15 March 2025)).

### 2.5. Real-Time Quantitative PCR (RT-qPCR) Validation

To validate the reliability of the transcriptome sequencing results, eight sex-related DEGs (*Gsdf*, *Eef1a1b*, *Dmrt1*, *Amh*, *Sox3*, *Figla*, *Foxo3a*, and *Gdf9*) showing significant sex-biased expression patterns and reported associations with reproductive processes in teleosts were selected for RT-qPCR analysis. Gene-specific primers were designed using Primer-BLAST (NCBI) based on the reference transcript sequences of *S. argus*, and the primer sequences are provided in Table S1. *β-actin* was used as the internal reference gene for normalization. The Ct values of *β-actin* were examined across ovary and testis samples, and consistent amplification was observed among biological replicates within each group. RT-qPCR was performed using a SYBR Green-based detection system on a LightCycler^®^ 96 Instrument (Roche Diagnostics, Mannheim, Germany). Each reaction was performed in a 20 μL volume containing 10 μL of SYBR Green Master Mix, 0.5 μL of each primer, 2 μL of a diluted cDNA template, and 7 μL of nuclease-free water. The thermal cycling program was: initial denaturation at 95 °C for 1 min, followed by 40 cycles of 95 °C for 30 s and 60 °C for 30 s. Melting curve analysis was performed after amplification to confirm the specificity of PCR products. The relative expression levels of target genes were calculated using the 2^−ΔΔCt^ method. Primer amplification efficiencies were evaluated using serially diluted cDNA templates, and the corresponding information is provided in Table S1. Data are presented as mean ± standard error of the mean (SEM) from three biological replicates. Statistical differences between ovary and testis groups were analyzed using Student’s *t*-test, with *p* < 0.05 considered statistically significant.

### 2.6. Validation of AS Events by RT-PCR

To confirm the presence of candidate alternative splicing (AS) isoforms, RT-PCR was performed using the PrimeScript RT Reagent Kit with gDNA Eraser and Premix Ex Taq II (TaKaRa Bio Inc., Kusatsu, Japan). Three biological replicates of ovaries and testes were used for RT-PCR validation. Isoform-specific primers targeting flanking exons or splice junctions were designed using NCBI Primer-BLAST online tool (https://www.ncbi.nlm.nih.gov/tools/primer-blast/; accessed on 15 February 2025) (Table S1). PCR amplification was performed in a 20 μL reaction mixture containing 10 μL of Premix Ex Taq II, 0.4 μL of each primer, 1 μL of a cDNA template, and 8.2 μL of nuclease-free water. The PCR amplification program was as follows: initial denaturation at 95 °C for 3 min, followed by 35 cycles of 95 °C for 30 s, 58–60 °C for 30 s, and 72 °C for 30 s, with a final extension at 72 °C for 5 min. PCR products were analyzed by 2% agarose gel electrophoresis and visualized under UV illumination.

### 2.7. Three-Dimensional Protein Structure Prediction

To assess the potential functional impact of AS events, protein domains were predicted using InterPro. The three-dimensional (3D) structures of the proteins encoded by both original and differentially spliced transcript isoforms were predicted using AlphaFold [22]. The PyMOL Molecular Graphics System (Schrödinger, New York, NY, USA) was utilized for the visualization of the predicted structures (http://www.pymol.org/).

### 2.8. Statistical Analysis and Data Visualization

Statistical analyses were performed using R software (v4.3.0, R Core Team) and GraphPad Prism (v8.0.2). Data visualization, including heatmaps and scatter plots, was conducted using the ggplot2 and pheatmap packages in R.

## 3. Results

### 3.1. Sequencing Quality Assessment of Full-Length Transcriptome

Full-length transcriptome sequencing was performed on gonadal tissues collected from three adult ovaries and three adult testes of *S. argus* using the Oxford Nanopore Technologies (ONT) platform. On average, 7.30 Gb of clean data were generated per sample, with an overall mean N50 read length of 1502 bp, indicating good sequencing performance across all samples (Table 1). After read correction, genome alignment, and transcript collapsing, a total of 63,000 non-redundant consensus transcripts were identified across all samples. Among them, an average of 23,227 and 28,452 transcript isoforms were detected in the ovaries and testis, respectively. The consensus transcripts had an N50 length of 2057 bp, an average length of 1647 bp, and a maximum length of 8036 bp (Table 2).

### 3.2. Analysis of Differentially Expressed Genes (DEGs) in the Gonads of S. argus

**Figure 1:**
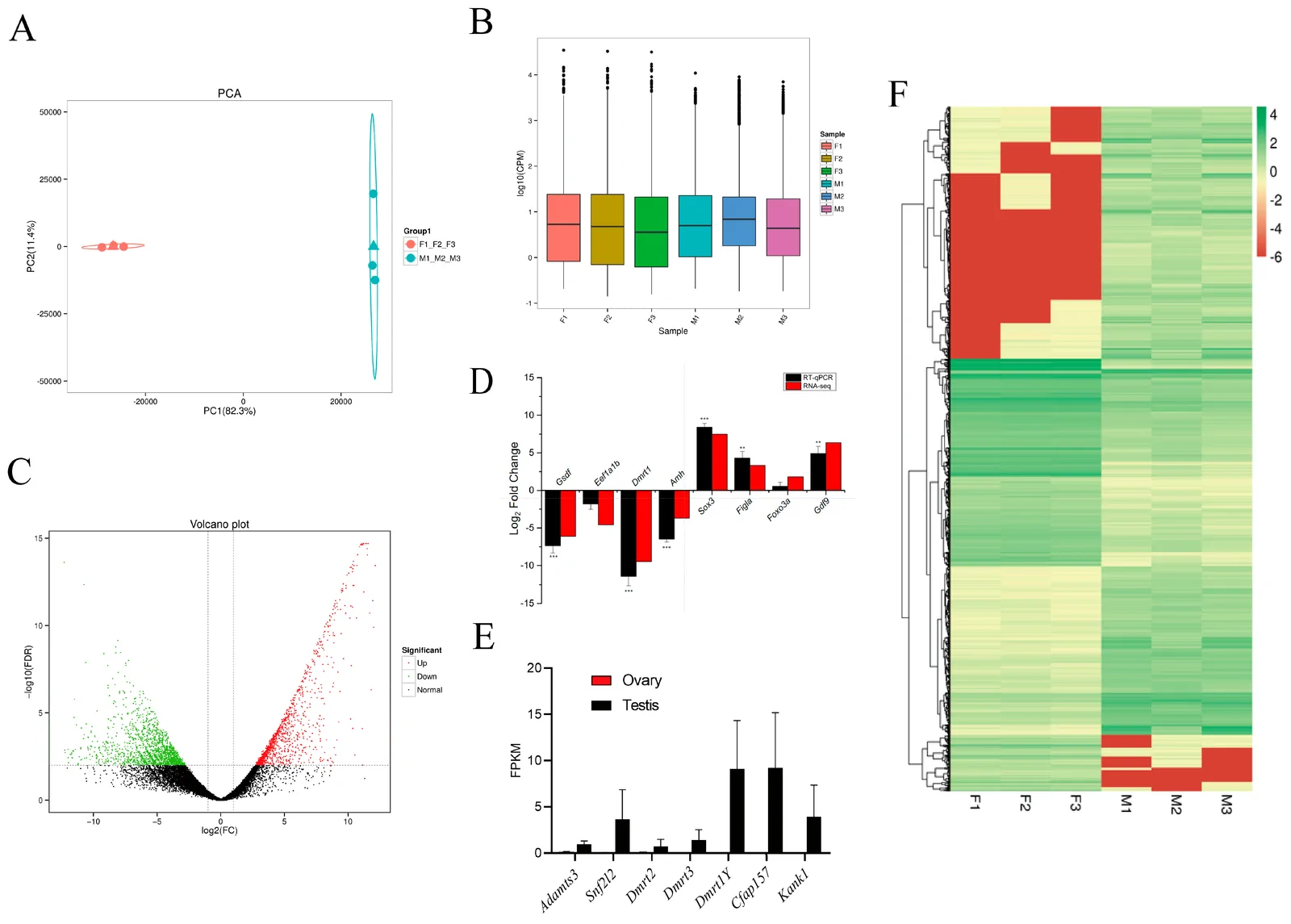
DEGs between male and female gonads of *S. argus* based on ONT full-length transcriptome sequencing. (**A**) Clustering of male (blue circles) and female (red circles) samples based on gonadal transcriptome profiles by principal component analysis (PCA). Each circle represents an individual biological replicate (*n* = 3 per group), and triangles indicate the centroid of each group in the PCA space. (**B**) Box plots of normalized gene expression values (log10-transformed) in male and female gonads. The median expression level is indicated by the horizontal line within each box. (**C**) Volcano plot of gene expression differences. Red dots represent genes upregulated in the testis; green dots represent genes upregulated in the ovary. (**D**) RT-qPCR analysis of selected sex-related DEGs identified from RNA-seq. Log_2_FC values represent ovary versus testis expression levels (log_2_[ovary/testis]). Black bars indicate RT-qPCR results, and red bars indicate RNA-seq results. Data are presented as mean ± SEM (*n* = 3 biological replicates). Statistical significance of RT-qPCR data was determined using Student’s *t*-test based on ΔCt values. Asterisks indicate significant differences (** *p* < 0.01; *** *p* < 0.001). (**E**) Expression levels of genes in the sex-determining region (SDR), including *Dmrt1Y*, based on male reference genome mapping. (**F**) Heatmap of hierarchical clustering of differentially expressed genes (DEGs).

Global expression patterns were visualized using normalized gene expression values. Principal component analysis (PCA) revealed distinct clustering of samples according to sex, with biological replicates from the ovary and testis grouping separately (Figure 1A). The distribution of normalized expression values showed a slightly higher median expression level in the testis compared with the ovary (Figure 1B). Using the criteria of |log2 fold change| > 2.0 and FDR < 0.05, a total of 3333 DEGs were identified between the gonads, including 1269 genes upregulated in the testis and 2064 genes upregulated in the ovary (Figure 1C; Table S2). To assess the consistency of expression patterns identified by RNA-seq, eight representative sex-biased DEGs were selected for RT-qPCR analysis. The RT-qPCR results showed expression trends consistent with those observed in the transcriptome dataset (Figure 1D). By aligning transcriptomic reads to the male reference genome [15], genes located in the reported sex-determining region, including the candidate sex-determining gene Dmrt1Y, exhibited testis-biased expression patterns (Figure 1E). Hierarchical clustering analysis further demonstrated distinct sex-biased expression patterns among DEGs in ovaries and testes (Figure 1F).

### 3.3. Alternative Splicing (AS) Analysis in Gonads of S. argus

**Figure 2:**
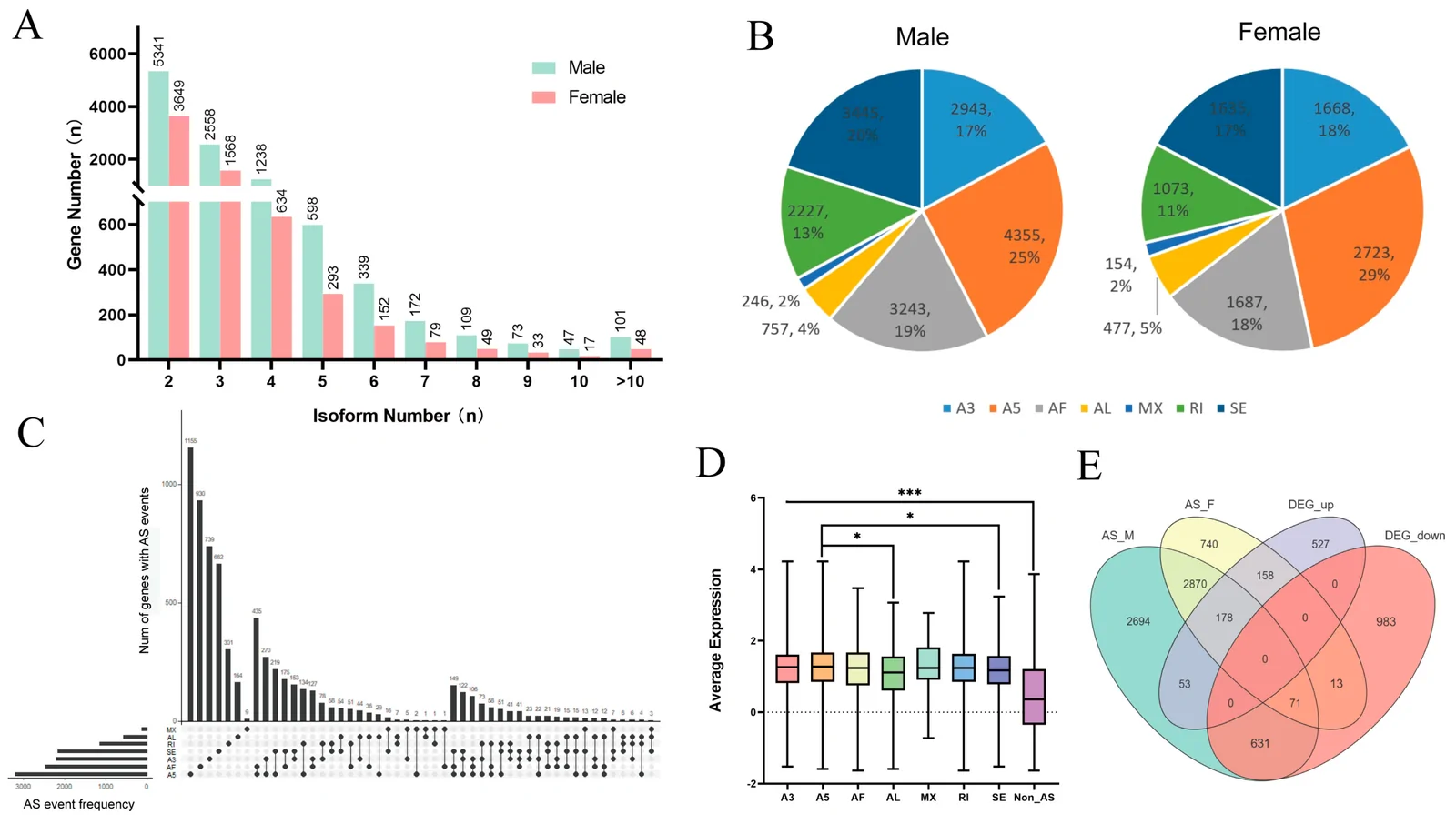
Identification and characterization of AS events of *S. argus*. (**A**) Isoform numbers of expressed genes and ratio of gene numbers (male relative to female) in gonads of *S. argus*. (**B**) Number and proportion of AS events in the gonads of *S. argus*. (**C**) UpSet plot showing the interactions among seven AS event types and the associated genes. SE, exon skipping; MX, mutually exclusive exons; RI, retained introns; AF, alternative first exon; AL, alternative last exon; A5, alternative 5′ splice site; A3, alternative 3′ splice site. (**D**) Distribution of average expression values for genes associated with different AS event types and genes without detectable AS events. Statistical significance is indicated as follows: ns, not significant; * *p* < 0.05; *** *p* < 0.001. (**E**) Venn diagram showing the overlap between DEGs and AS-associated genes.

A total of 6522 and 10,576 genes expressing two or more transcript isoforms were identified in ovaries and testes, respectively (Figure 2A). A total of 17,216 AS events were detected in testes, with alternative 5′ splice sites (A5) representing the most abundant AS type (approximately 25%), whereas mutually exclusive exons (MX) were the least frequent (approximately 1.4%). In ovaries, 9417 AS events were identified, displaying a similar distribution of AS types but a lower overall frequency than that observed in males (Figure 2B; Table S3). To examine the combinatorial patterns of AS, an UpSet analysis was performed on the 11,854 AS-associated genes. Approximately one-third of these genes (3960/11,854) were associated with a single AS type, whereas the remaining genes exhibited two or more AS types, indicating widespread combinatorial splicing patterns in adult gonads (Figure 2C). Genes associated with AS events generally exhibited higher normalized expression levels than genes without detectable AS events (Figure 2D). Comparison of AS-associated genes with differentially expressed genes (DEGs) revealed 1104 overlapping genes, suggesting that both alternative splicing and differential expression contribute to the transcriptomic differences between male and female gonads (Figure 2E).

### 3.4. AS Analysis of Sex-Related Genes in the Gonads

**Figure 3:**
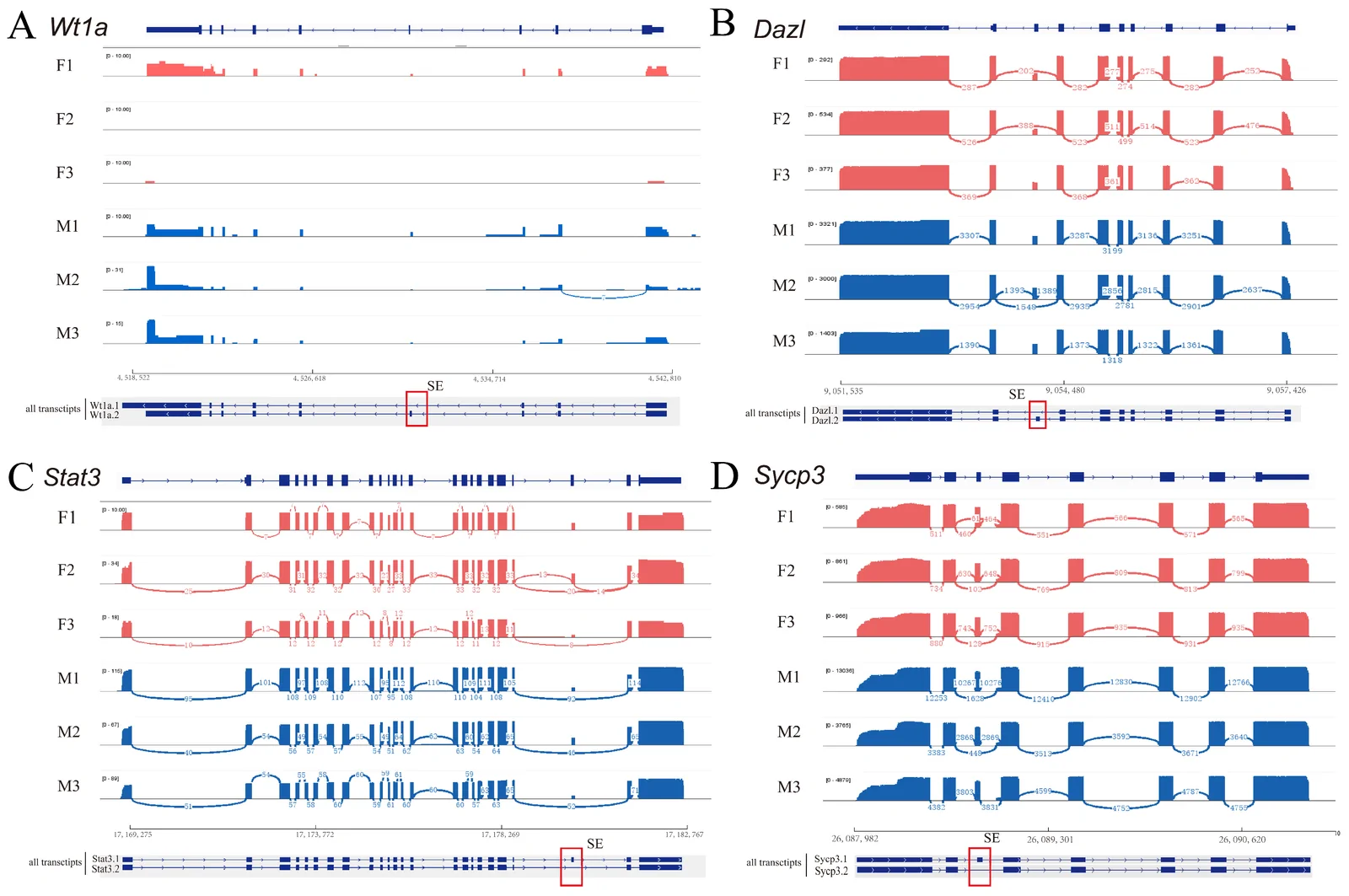
Transcript structures and alternative splicing patterns of representative sex-related DEGs in *S. argus* gonads. Sashimi plots show exon usage and splice junction reads in female and male gonads. (**A**) *Wt1a* exhibits exon 4 skipping. (**B**) *Dazl* exhibits exon 8 skipping. (**C**) *Sycp3* exhibits exon 3 skipping. (**D**) *Stat3* exhibits exon 22 skipping. Arrows indicate the direction of transcription.

The candidate sex-determining gene *Dmrt1Y* and its X-linked allele *Dmrt1△X* each exhibited a single predominant transcript structure, and no detectable AS events were identified in either gene in the adult gonads examined. To further characterize representative AS events in sex-related genes, four genes (*Wt1a*, *Dazl*, *Sycp3*, and *Stat3*) were selected for transcript structure visualization using Sashimi plots. *Wt1a* exhibited two predominant isoforms differing by exon 4 skipping (84 bp) (Figure 3A). *Dazl* exhibited exon 8 skipping (51 bp) (Figure 3B). *Sycp3* showed exon 3 skipping (35 bp) (Figure 3C), which introduces a frameshift as the skipped exon length is not divisible by three. *Stat3* exhibited exon 22 skipping (60 bp) (Figure 3D). These AS events were consistently detected in both ovaries and testes. To evaluate the potential structural consequences of these AS events, protein domain organization and three-dimensional protein structures were predicted for each isoform (Figure S1). The SE event in *Wt1a* is predicted to result in the loss of a conserved protein domain, whereas the AS events in *Dazl* and *Stat3* occur in-frame and are not predicted to cause substantial changes in conserved domains. The frameshift in *Sycp3* potentially alters the downstream amino acid sequence and C-terminal protein structure (Figure S1).

### 3.5. Differential Alternative Splicing (DAS) Analysis of Gonads

**Figure 4:**
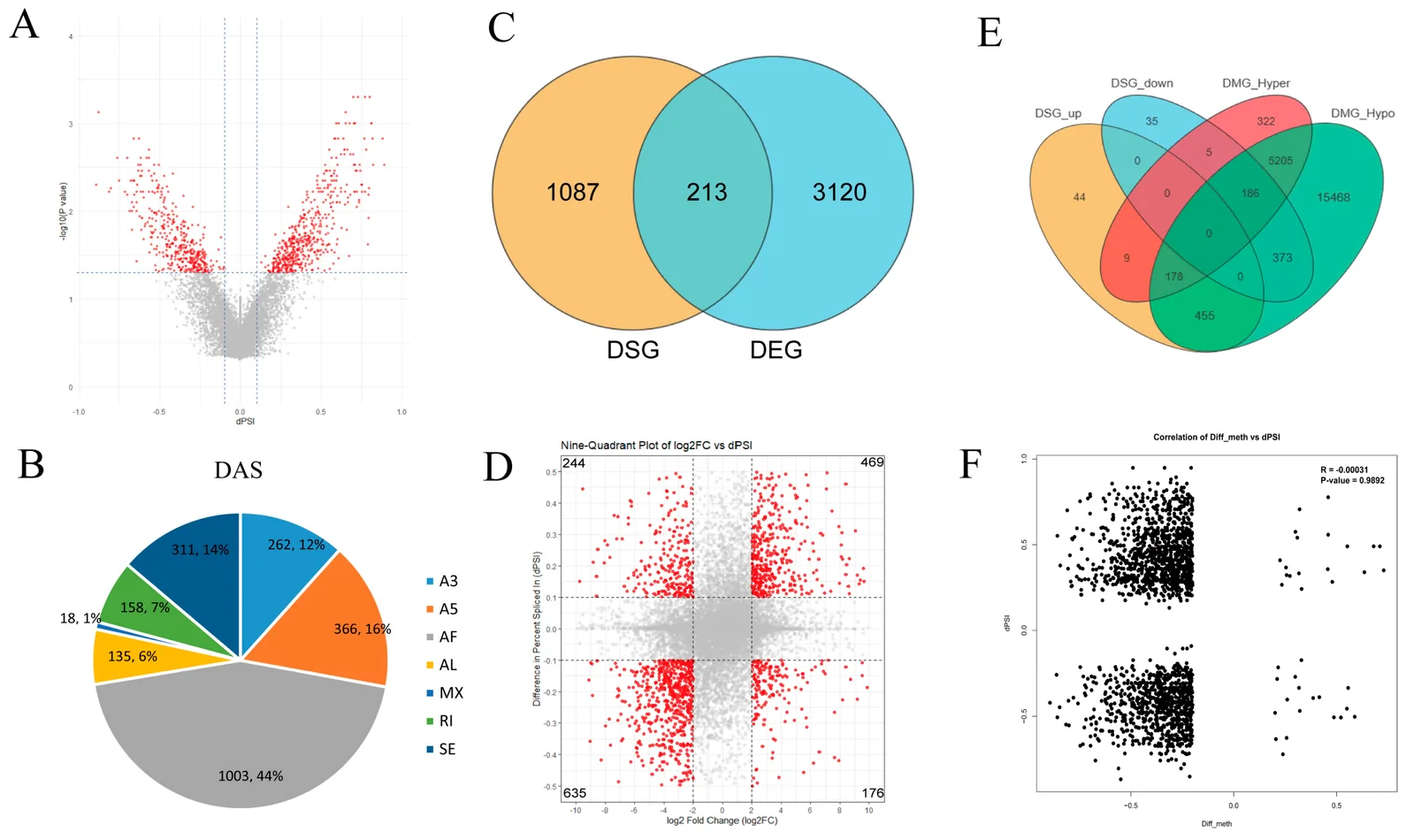
DAS identification and analysis in the gonads of *S. argus*. (**A**) Volcano plot illustrating the significant DAS events (dPSI: difference in Percent Spliced-In). (**B**) Number and proportion of the seven distinct AS event types among the identified DAS events. (**C**) Venn diagram showing the overlap between DEGs and DSGs. (**D**) Quadrant scatter plot showing DEGs that underwent DAS in the gonads, with each dot representing a single gene. (**E**) Venn diagram showing the overlap between DSGs and differentially methylated genes (DMGs). (**F**) Correlation between DNA methylation differences and alternative splicing variations in the gonads.

DAS events between the ovaries and testes were identified using thresholds of |dPSI| > 0.1 and an FDR-adjusted *p*-value < 0.05. A total of 2253 DAS events were detected (Figure 4A; Table S4), with the alternative first exon (AF; 44.51%), alternative 5′ splice site (A5; 16.25%), and exon skipping (SE; 13.80%) being the most predominant among the seven types identified (Figure 4B). These events mapped to 1300 differentially spliced genes (DSGs), of which 213 were also differentially expressed (Figure 4C). Quadrant plot analysis based on gene-level log2FC and dPSI values revealed a positive correlation between gene expression changes and alternative splicing changes in 1104 genes (Figure 4D). To explore the epigenetic regulation of alternative splicing, we integrated our DSGs with previously reported differentially methylated genes (DMGs) in *S. argus* gonads (Jiao et al., 2024) [20]. Although 1206 DSGs overlapped with DMGs (Figure 4E), no significant correlation was detected between gene-level DNA methylation differences (Diff_meth) and dPSI values (Pearson correlation, R = −0.00031; Figure 4F). DNA methylation profiles of representative overlapping genes, including *Wt1a*, *Dazl, Sycp3*, *Stat3*, *Ncoa5*, *Tp53*, and *Fancl*, are presented in Figures S3–S9, showing differential methylation patterns between ovaries and testes.

### 3.6. Functional Comparison and Enrichment Analysis of DEGs and DSGs

**Figure 5:**
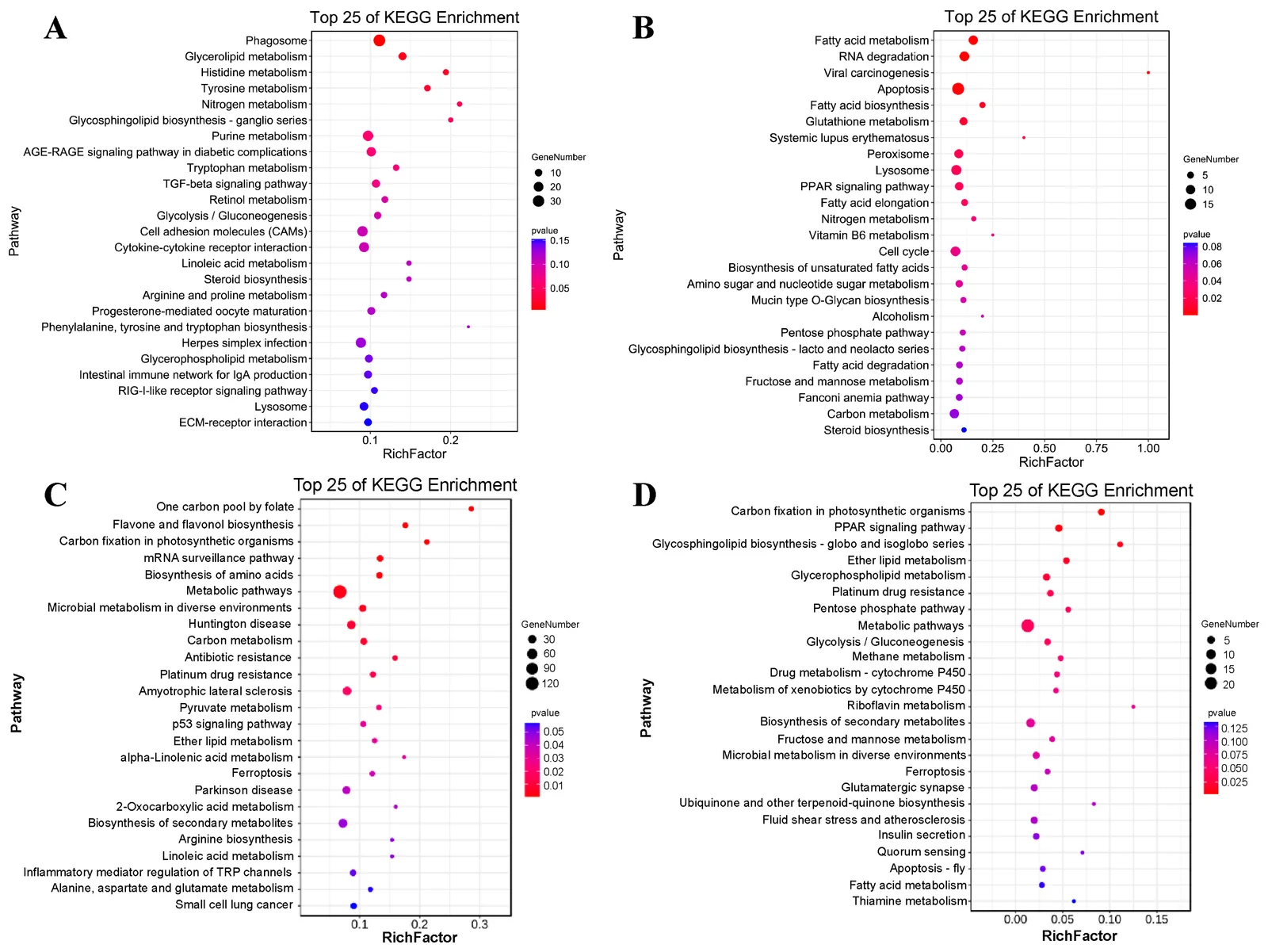
KEGG pathway enrichment analysis of DEGs and DSGs in the gonads of *S. argus*. (**A**) Significant KEGG enrichment pathways for ovary-biased DEGs. (**B**) Significant KEGG enrichment pathways for testis-biased DEGs. (**C**) Significant KEGG enrichment pathways for the identified DSGs. (**D**) Significant KEGG enrichment pathways for the overlapping genes between DEGs and DSGs. The diameter of the circles represents the number of genes associated with each pathway. The color gradient, ranging from red to blue, indicates the significance level based on *p*-value.

**Figure 6:**
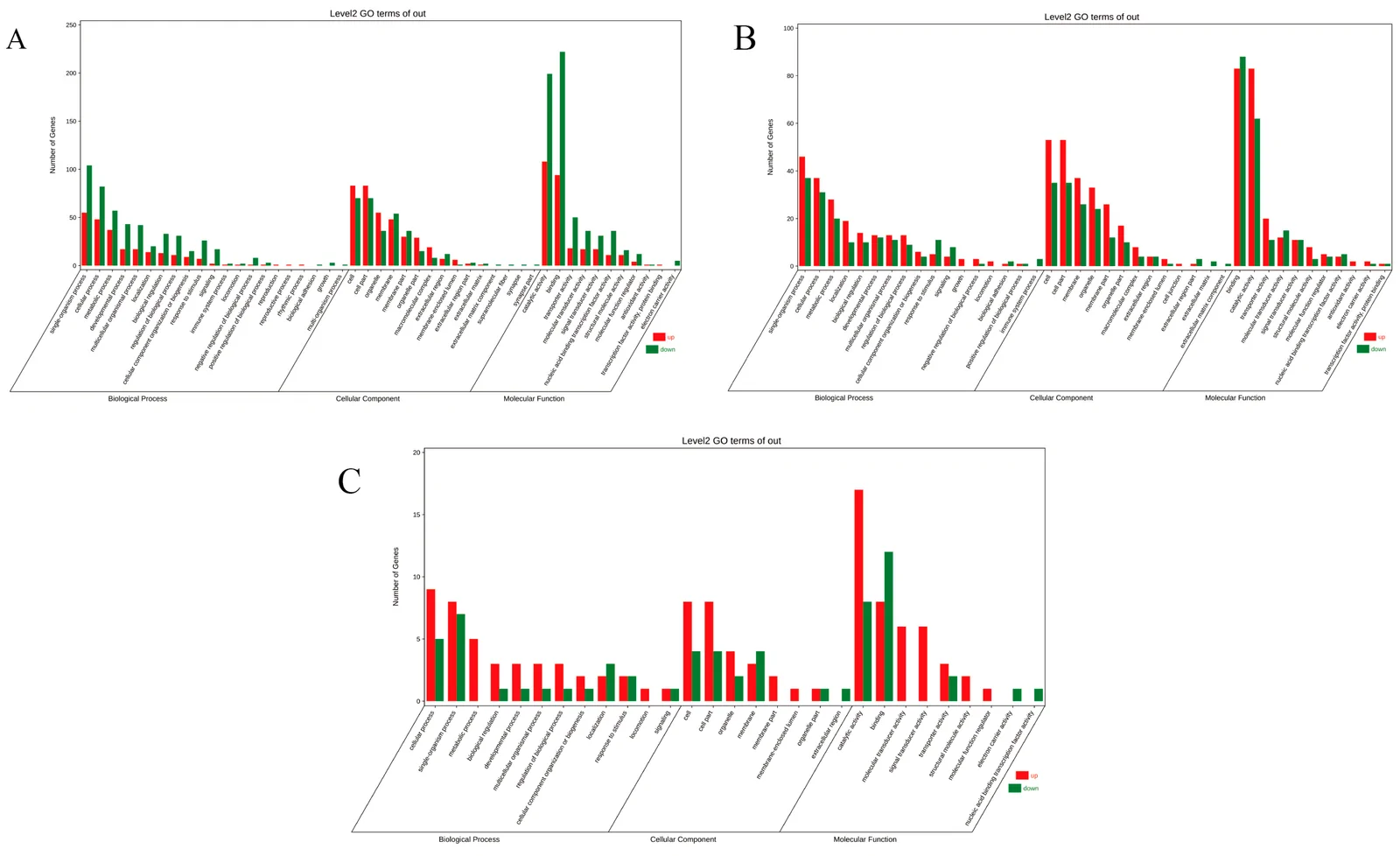
GO enrichment analysis of genes in *S. argus* gonads. (**A**) Significantly enriched GO terms for DEGs. (**B**) Significantly enriched GO terms for DSGs. (**C**) Significantly enriched GO terms for genes overlapping between DEGs and DSGs. The horizontal axis indicates GO term names, and the vertical axis indicates the number of genes assigned to each term.

To investigate the functional characteristics of DEGs and DSGs in the gonads of *S. argus*, KEGG and GO enrichment analyses were performed. Among the 3333 DEGs, the 2064 ovary-biased genes were significantly enriched in oocyte meiosis, progesterone-mediated oocyte maturation, and PI3K-Akt signaling pathways (Figure 5A; Table S5). In contrast, the 1269 testis-biased DEGs were primarily enriched in cell cycle, DNA replication, and spliceosome pathways (Figure 5B; Table S5). The 1300 DSGs showed distinct enrichment patterns, including endocytosis, focal adhesion, and regulation of the actin cytoskeleton (Figure 5C; Table S6). Among the 213 genes shared between DEGs and DSGs, metabolic pathways represented the most significantly enriched KEGG category (Figure 5D; Table S7). GO enrichment analysis revealed different functional characteristics between DEGs and DSGs. DEGs were mainly enriched in catalytic activity and microtubule-based processes (Figure 6A; Table S8), whereas DSGs were enriched in protein binding, intracellular transport, organelle organization, and establishment of localization (Figure 6B; Table S9). The overlapping genes were significantly enriched in protein phosphorylation and transition metal ion binding (Figure 6C; Table S10).

### 3.7. Characterization of Sex-Associated Splice Isoforms in Key Gonadal Genes

To further investigate the potential consequences of differential alternative splicing, three sex-related DSGs (*Ncoa5*, *Tp53*, and *Fancl*) were selected for detailed analysis. Sashimi plots were used to characterize transcript structures, and RT-PCR analysis was performed to confirm the presence of selected splice variants. Because conventional RT-PCR cannot quantitatively assess isoform usage, isoform abundance patterns were evaluated based on transcript abundance derived from the full-length transcriptome data.

**Figure 7:**
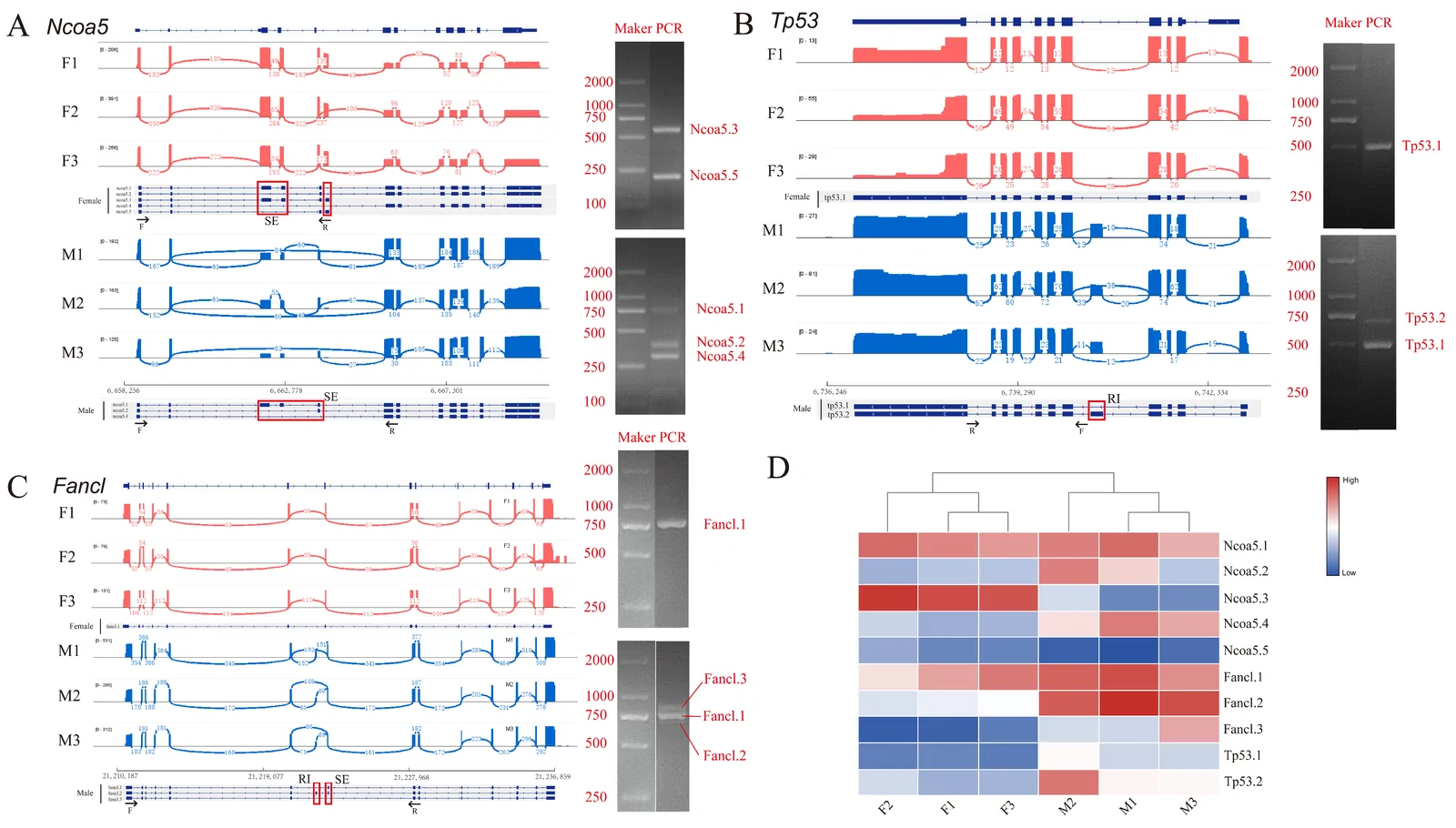
Full-length transcript structures and RT-PCR validation of representative differentially spliced genes (DSGs). Sashimi plots showing RNA-seq-supported transcript structures and alternative splicing events in ovaries and testes are presented. Red boxes highlight the regions containing the identified alternative splicing events. (**A**) *Ncoa5*, (**B**) *Tp53*, and (**C**) *Fancl*. F1–F3 and M1–M3 represent three biological replicates of ovary and testis samples, respectively. Arrows indicate the direction of gene transcription. Selected splice variants identified from full-length transcriptome sequencing were validated by RT-PCR. Representative RT-PCR amplification products are shown, with the corresponding transcript isoforms indicated. (**D**) Heatmap depicting the transcript abundance profiles of identified isoforms of these genes based on full-length transcriptome sequencing.

*Ncoa5* generated five transcript isoforms in the gonads. Sashimi plot analysis characterized multiple exon-skipping events in this gene, and RT-PCR further confirmed the presence of the corresponding splice variants (Figure 7A). Transcript abundance analysis based on the full-length transcriptome data showed that the full-length isoform (*Ncoa5.1*) exhibited comparable expression levels between sexes, whereas two truncated isoforms, *Ncoa5.2* (skipping exons 3 [273 bp] and 4 [108 bp]) and *Ncoa5.4* (skipping exons 3, 4, and 5 [63 bp]), were predominantly expressed in the testis (Figure 7D). Protein structure prediction indicated that these exon-skipping events resulted in the predicted loss of the RNA recognition motif (RRM) domain (Figure S2A). In contrast, *Ncoa5.3* and *Ncoa5.5* were ovary-associated isoforms generated through alternative splicing downstream of exon 6, and *Ncoa5.5* lacked a complete open reading frame.

*Tp53* exhibited a testis-associated intron retention event involving partial retention of intron 4 (191 bp), generating the *Tp53.2* isoform (Figure 7B). RT-PCR further supported the presence of this transcript isoform. This intron retention event introduced a frameshift and was predicted to result in partial loss of the *p53* DNA-binding domain according to protein structure prediction (Figure S2B). The major transcript isoform, *Tp53.1*, did not show marked sex-biased abundance (Figure 7D).

*Fancl* displayed two alternative splicing events identified from the full-length transcriptome data and supported by Sashimi plot analysis, generating two testis-associated splice isoforms that were further confirmed by RT-PCR (Figure 7C). These included partial retention of intron 6 (75 bp), generating *Fancl.2*, and skipping of exon 7 (69 bp), generating *Fancl.3*. Neither event caused a frameshift; however, both were predicted to result in partial loss of the UBC-like domain 2 (Figure S2C). Interestingly, although overall *Fancl* expression was higher in the testis, the full-length isoform (*Fancl.1*) was more abundant in the ovary (Figure 7D).

## 4. Discussion

Understanding the molecular mechanisms associated with adult gonadal maintenance and gametogenesis is important for providing insights into sex-control breeding strategies in *S. argus* aquaculture. While alternative splicing (AS) is increasingly recognized as an important post-transcriptional regulatory process in teleost reproduction, its characteristics and potential contributions in *S. argus* remain largely unexplored [23,24,25]. By leveraging Oxford Nanopore Technologies (ONT), this study circumvented the limitations of short-read assembly, enabling the accurate identification of full-length transcript isoforms and global AS events in stage IV gonads. These findings provide a valuable resource for investigating post-transcriptional regulation associated with adult gonadal maintenance and gametogenesis.

In this study, adult testes exhibited higher alternative splicing activity and transcript diversity than ovaries. Similar patterns have been reported in mammals, where testes display highly complex transcriptomic profiles [26,27]. This similarity suggests that increased transcriptomic complexity may be associated with the diverse regulatory requirements of spermatogenic tissues across vertebrates.

Key sex-related genes exhibited robust expression dimorphism: *Dmrt1Y*, *Dmrt1△X*, *Amh*, and *Gsdf* were testis-biased, whereas *Figla*, *Gdf9*, *Sox3*, and *Foxo3a* were ovary-biased, consistent with previous reports in *S. argus* [19,28]. These reciprocal expression patterns support the reliability of our dataset and are consistent with the reported involvement of these genes in teleost gonadal development. Beyond transcriptional differences, alternative splicing (AS) represents an additional post-transcriptional regulatory process in the gonads of *S. argus*. The number of AS events was markedly higher in the testis than in the ovary, indicating pronounced sex-biased splicing patterns. This pattern is consistent with that observed in other teleosts, such as zebrafish and Chinese tongue sole [26,29]. In addition, genes undergoing AS generally exhibited higher expression levels than non-spliced genes. This association suggests that alternatively spliced genes may contribute to transcriptomic complexity in the gonads of *S. argus*. However, the biological significance of the higher prevalence of AS events in testes remains unclear and requires further investigation.

Furthermore, although epigenetic modifications, particularly DNA methylation, have been reported to influence alternative splicing regulation in mammals [30,31], our integrative analysis combining DAS results with previously published gonadal methylation data [20] revealed no significant genome-wide correlation between differentially methylated genes and differential alternative splicing events in *S. argus* gonads. Consistently, the DNA methylation profiles of representative sex-related genes (Figures S3–S9) showed no obvious sex-specific methylation differences near the identified splice sites. These results suggest that DNA methylation variation alone may not fully explain the observed sex-biased alternative splicing patterns in adult gonads of *S. argus*. Other regulatory mechanisms, including potential contributions from RNA-binding proteins or spliceosomal components, may also participate in shaping sex-specific splicing patterns, although this possibility requires further investigation. In addition, the limited overlap between DSGs and DEGs (213 out of 1300 DSGs) highlights that alternative splicing and transcriptional regulation represent partially independent regulatory layers in adult gonads. Functional enrichment analyses further supported this distinction: DEGs were mainly associated with processes including cell cycle regulation and metabolic pathways, whereas DSGs were enriched in functions related to intracellular transport, protein binding, and cytoskeletal organization. The overlapping genes between these two regulatory groups were enriched in protein phosphorylation and signal transduction pathways, suggesting that a subset of genes may undergo coordinated regulation at both transcriptional and post-transcriptional levels during adult gonadal maintenance and gametogenesis.

A notable observation in this study was the absence of detectable alternative splicing events in the candidate sex-related genes *Dmrt1Y* and its X-linked homolog *Dmrt1△X*. As previously reported sex-related genes in *S. argus*, both genes exhibited a single predominant transcript structure in the adult gonads analyzed here. In contrast, other sex-related genes, including *Wt1a*, *Dazl*, *Sycp3*, and *Stat3*, displayed clear alternative splicing patterns. Specifically, exon-skipping (SE) events were detected in all four genes, resulting in different transcript structures. For instance, the skipped exon in *Sycp3* introduced a frameshift and was predicted to alter the downstream amino acid sequence and protein structure. Whether this structural alteration affects *Sycp3* function requires further experimental validation [32,33]. Conversely, the SE events in *Wt1a*, *Dazl*, and *Stat3* preserved the reading frame. Protein structure prediction indicated that the skipped exon in *Wt1a* may result in partial loss of a predicted functional region, although the biological consequences of this change remain unclear [34,35]. Meanwhile, the in-frame variants of *Dazl* and *Stat3* retained their major annotated domains, suggesting that these events may contribute to transcript diversity without disrupting core protein domains [36,37,38]. Overall, these observations indicate that, in the adult gonads examined here, *Dmrt1Y* and *Dmrt1△X* exhibited relatively conserved transcript structures, whereas other sex-related genes displayed diverse AS patterns. Whether these sex-related differences in alternative splicing are specific to adult gonads or represent developmental stage-dependent regulatory mechanisms remains to be investigated through comparative analyses across different gonadal developmental stages.

Beyond these shared splicing events, several differentially spliced genes—particularly *Ncoa5*, *Tp53*, and *Fancl*—exhibited pronounced sex-specific isoform usage. These genes have been previously associated with reproductive or germ-cell-related processes in vertebrates. To further explore their potential biological relevance, we integrated our findings with previously published single-cell RNA-seq data [39], which showed that these genes are predominantly expressed in germ-cell populations, including spermatogonia and primary spermatocytes. This expression pattern provides additional context for their potential association with germ-cell-related processes. Structurally, the identified sex-specific AS events were predicted to affect regions corresponding to conserved functional domains. Previous studies have demonstrated that alternative splicing can generate functionally distinct protein isoforms in reproductive regulators. For example, different splice variants of *Dmrt1*, a conserved sex-related gene in vertebrates, have been reported to affect transcript structures and potentially influence its regulatory functions during gonadal development [12,13]. These findings suggest that sex-biased AS events identified in *S. argus* may represent potential mechanisms for diversifying protein functions rather than merely reflecting transcriptional variation. *Ncoa5* has been reported to participate in estrogen signaling and ovarian-related processes [40]. In *S. argus*, we identified a testis-specific exon-skipping event in *Ncoa5*, which was predicted to result in loss of part of the RNA recognition motif (RRM). This structural alteration may influence the predicted protein configuration, although its functional significance remains to be experimentally determined. Conversely, the ovary-specific truncated isoforms (*Ncoa5.3* and *Ncoa5.5*) exhibited distinct predicted transcript structures. However, the specific cell types expressing these isoforms and their biological significance remain unknown. Future single-cell transcriptomic analysis of the *S. argus* ovary may help clarify the cellular context of these isoforms.

Furthermore, *Fancl* and *Tp53* are functionally linked to pathways involved in germ-cell survival, apoptosis, and DNA-damage responses [41], and *Fancl* has been reported to regulate *Tp53*-mediated apoptosis during teleost meiosis [42,43]. In the *S. argus* testis, AS events in *Fancl* were predicted to result in partial loss of the UBC-like domain 2. Because this domain has been reported to contribute to the assembly and activity of the Fanconi anemia core complex [44], this predicted structural alteration may affect the properties of the encoded isoform; however, its functional consequences require further experimental validation. Concurrently, *Tp53* exhibited a male-specific intron retention event that was predicted to alter the DNA-binding domain and generate a truncated isoform [45]. Although similar truncated *p53* isoforms have been reported to modulate canonical signaling by interacting with full-length proteins in other vertebrates [46,47], whether the *Tp53* isoform identified in *S. argus* exhibits similar properties remains unknown. Therefore, these sex-biased AS events in *Fancl* and *Tp53* may represent candidate post-transcriptional features associated with germ-cell-related processes in adult testes.

In conclusion, this study reveals that AS represents an important post-transcriptional regulatory layer accompanying transcriptional regulation in adult gonads. The extensive occurrence of sex-biased splicing, the limited overlap between DSGs and DEGs, and the structural diversification observed in key gonad-related genes collectively suggest that AS may contribute to transcriptomic diversity associated with adult gonadal maintenance and gametogenesis.

## 5. Conclusions

Using ONT long-read sequencing, we generated full-length transcriptomes of adult *S. argus* testes and ovaries, identifying 3333 DEGs and 2253 DAS events between sexes. Several gonad-related genes, including *Ncoa5*, *Tp53*, and *Fancl*, exhibited sex-biased AS patterns, with some isoforms predicted to affect regions corresponding to functional domains. Integration with previous single-cell transcriptomic data suggested that these genes are enriched in germ-cell populations. Together, these findings reveal extensive AS diversity in adult *S. argus* gonads and highlight the potential contribution of AS-mediated post-transcriptional regulation to gonadal maintenance and gametogenesis.

## Figures and Tables

**Table 1:** Statistics of clean data.

FileName	ReadNum	BaseNum	N50	MeanLength (bp)	MaxLength (bp)
F1	4,892,392	5,807,756,778	1388	1187	12,150
F2	7,178,745	8,946,726,430	1465	1246	11,808
F3	6,533,670	7,858,293,450	1396	1202	8240
M1	4,944,378	7,222,946,226	1771	1460	12,701
M2	5,626,199	6,682,197,057	1452	1187	14,249
M3	5,581,433	7,287,683,016	1542	1305	12,739

**Table 2:** Summary of transcript statistics.

FileName	ReadNum	BaseNum	N50	MeanLength (bp)	MaxLength (bp)
F1	20,832	34,099,402	2039	1636	7132
F2	26,530	45,545,161	2126	1716	7189
F3	22,318	34,871,325	1884	1562	4553
M1	27,622	50,999,569	2198	1846	7125
M2	27,737	45,786,649	2088	1650	7667
M3	29,996	49,329,242	2029	1644	8036
All	63,000	103,763,807	2057	1647	8036

## Data Availability

The original contributions presented in this study are included in the article/Supplementary Material. Further inquiries can be directed to the corresponding authors.

## Supplementary Materials

The following supporting information can be downloaded at https://www.mdpi.com/article/10.3390/biom16091270/s1, Figure S1. Protein prediction of sex-related DEGs. This figure includes protein domain predictions and 3D protein structure modeling for the following genes: (A) *Wt1a*, (B) *Dazl*, (C) *Sycp3*, and (D) *Stat3*; Figure S2. Protein prediction of sex-related DSGs. This figure includes protein domain predictions and 3D protein structure modeling for the following genes: (A) *Ncoa5*, (B) *Tp53*, and (C) *Fancl*; Figure S3. DNA methylation profiles of *Wt1a*. Yellow and blue background regions represent DMR regions. Yellow and blue indicate hypermethylation and hypomethylation, respectively, in males. Note: If not specified, the ovary was used as a control group. The expression levels in different samples are displayed on the right; Figure S4. DNA methylation profiles of *Dazl*; Figure S5. DNA methylation profiles of *Sycp3*; Figure S6. DNA methylation profiles of *Stat3*; Figure S7. DNA methylation profiles of *Ncoa5*; Figure S8. DNA methylation profiles of *Tp53*; Figure S9. DNA methylation profiles of *Fancl*; Figure S10. Cell-type-specific expression profiles of five selected differential alternative splicing genes in the adult testis of *S. argus*. Dot plot illustrating the expression distribution of *Ncoa5*, *Fancl*, *Tp53*, *Wt1a*, and *Dazl* across various testicular cell clusters based on scRNA-seq data. The size of each dot (pct.exp) indicates the proportion of cells within a specific cluster that express the target gene. The color gradient (avg.exp.scale) represents the average scaled expression level, ranging from blue (low expression) to red (high expression). Note the distinct compartmentalization: *Dazl*, *Tp53*, and *Fancl* are predominantly enriched in germ-cell lineages (e.g., spermatogonia and primary spermatocytes), whereas *Wt1a* exhibits strict somatic specificity in Sertoli cells; Table S1. Primer sequences used in this study; Table S2. DEGs identified in this study; Table S3. AS identified in this study; Table S4. DAS identified in this study (*p*-value < 0.05); Table S5. KEGG pathway enrichment analysis of the DEGs (*p*-value < 0.05); Table S6. GO analysis of DEGs; Table S7. KEGG pathway enrichment analysis of the DSGs (*p*-value < 0.05); Table S8. GO analysis of DSGs; Table S9. KEGG pathway enrichment analysis of the overlapping genes between DEGs and DSGs (top25); Table S10. GO analysis of overlapping genes between DEGs and DSGs.
