# Supplementary material for: Full-Length Transcriptome Comparison of Male and Female Gonads in Scatophagus argus Reveals Alternative Splicing Events
Source: Biomolecules. 2026 Sep 2;16(9):1270. doi: 10.3390/biom16091270 (PMC13604722; doi:10.3390/biom16091270)
Supplement: Supplementary file 1 [file biomolecules-16-01270-s001.zip › Supplementary figures.pdf]

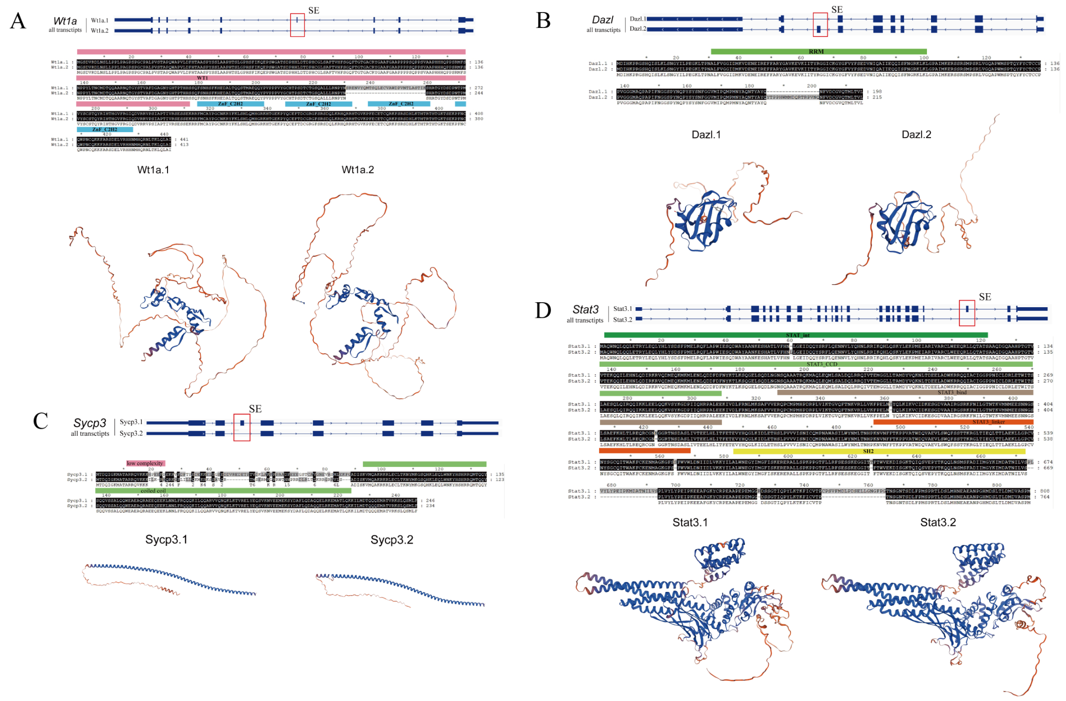

Figure S1. Protein prediction of sex-related DEGs. This figure includes protein domain predictions and 3D protein structure modeling for the following genes: (A) *Wt1a*, (B) *Dazl*, (C) *Sycp3*, and (D) *Stat3*.

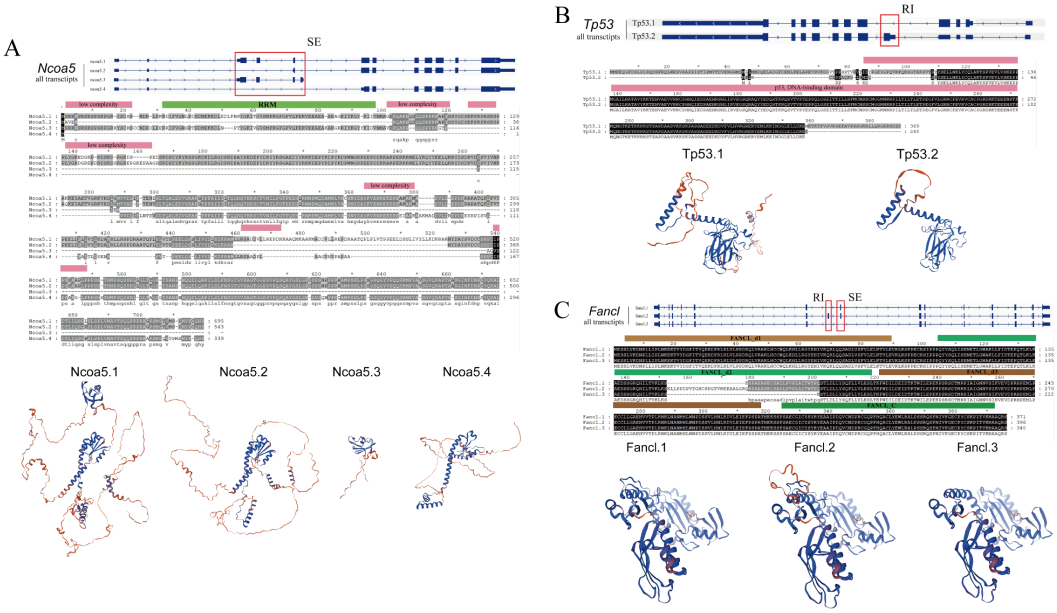

Figure S2. Protein prediction of sex-related DSGs. This figure includes protein domain predictions and 3D protein structure modeling for the following genes: (A) *Ncoa5*, (B) *Tp53*, and (C) *Fancl*.

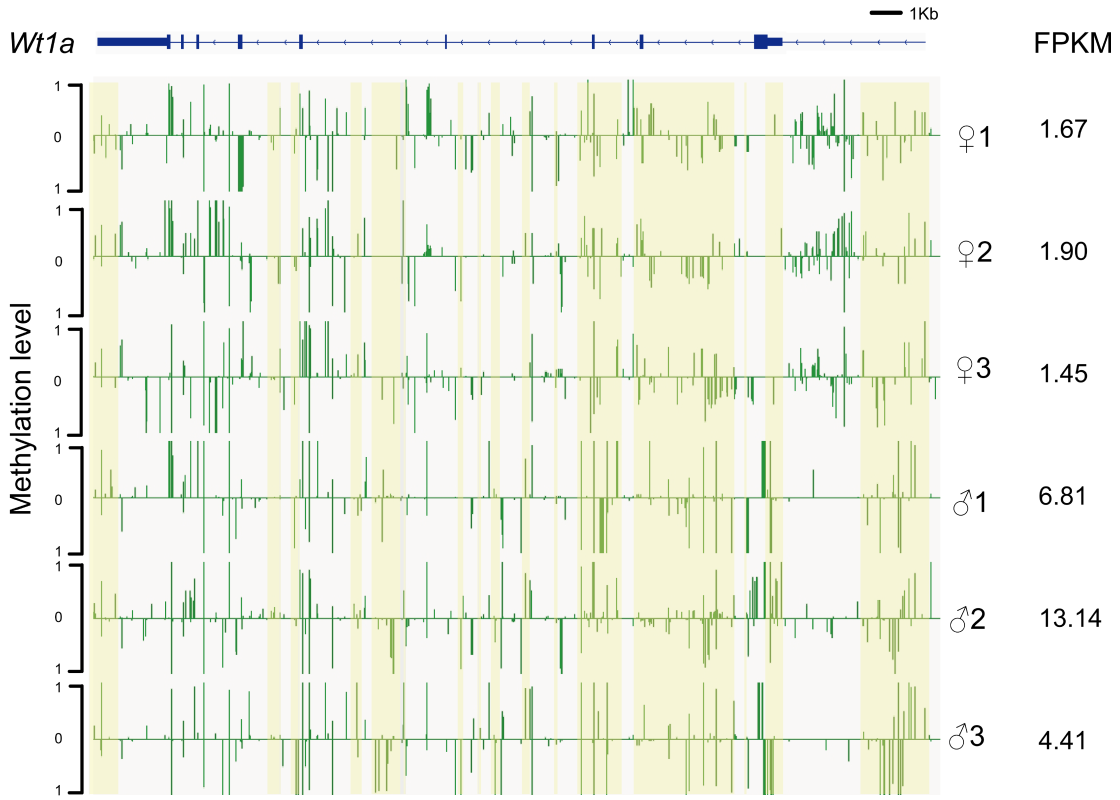

Figure S3. DNA methylation profiles of *Wt1a*. Yellow and blue background regions represent DMR regions. Yellow and blue indicate hypermethylation and hypomethylation, respectively, in males. Note: If not specified, the ovary was used as a control group. The expression levels in different samples are displayed on the right.

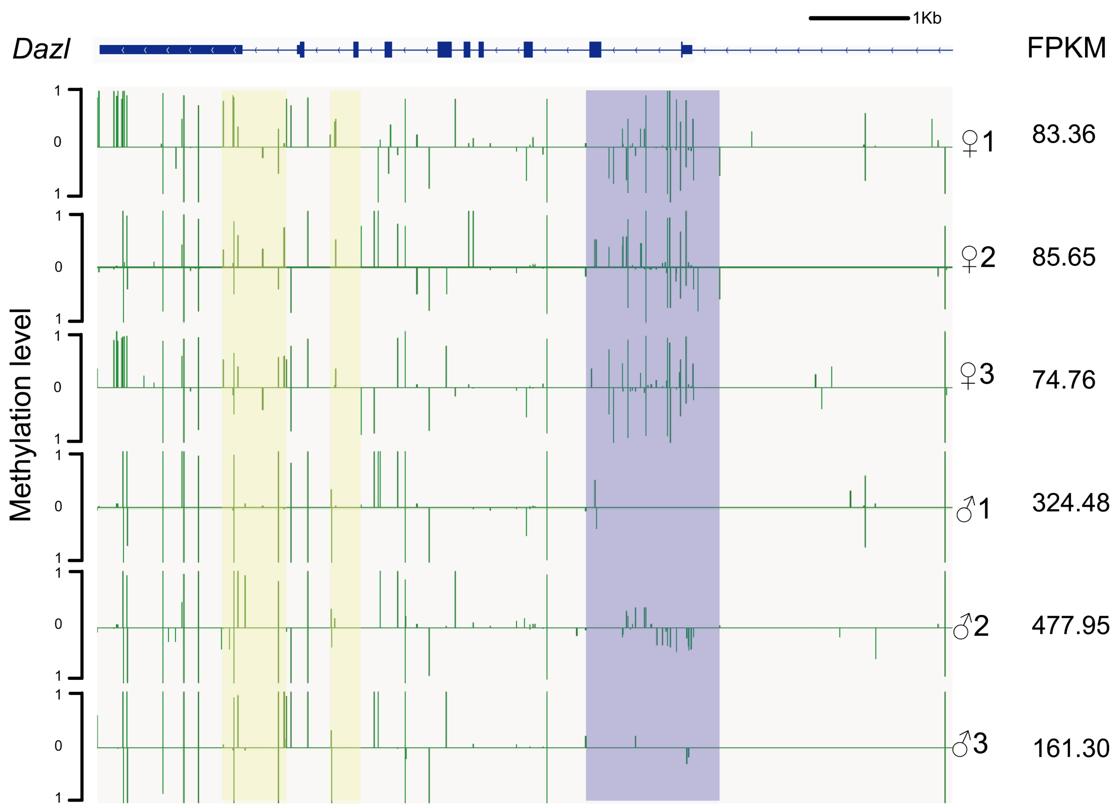

Figure S4. DNA methylation profiles of *Dazl*.

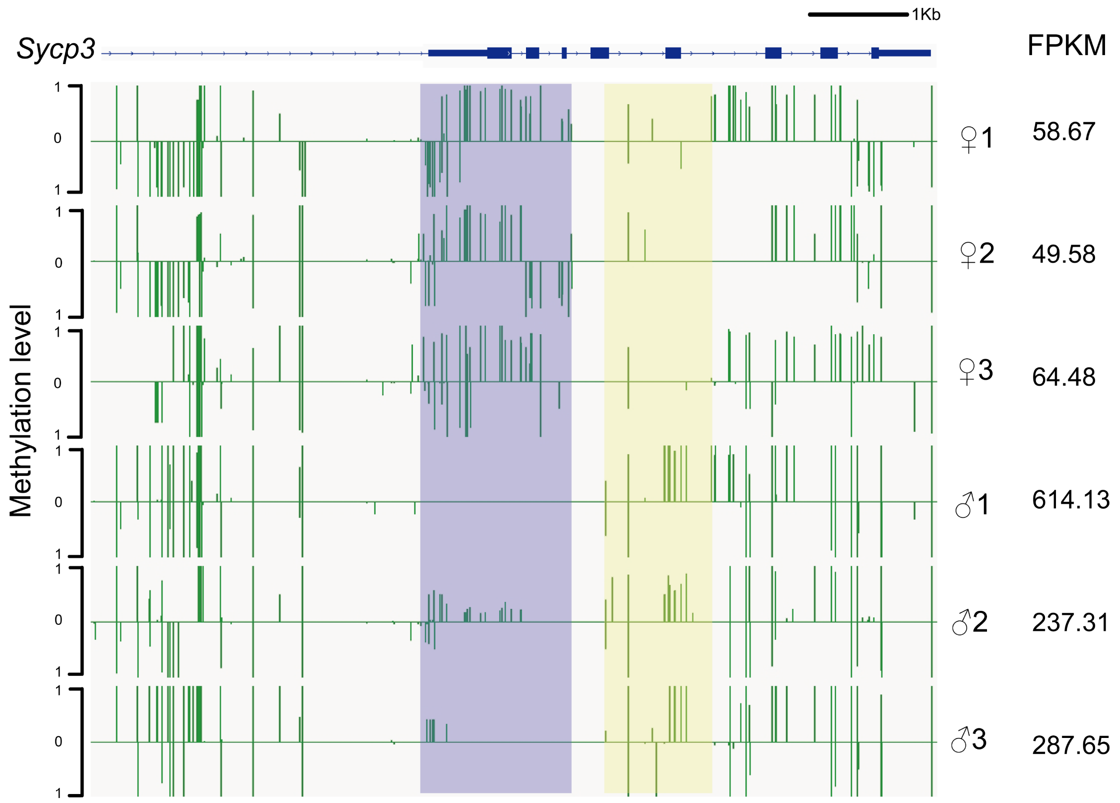

Figure S5. DNA methylation profiles of *Sycp3*.

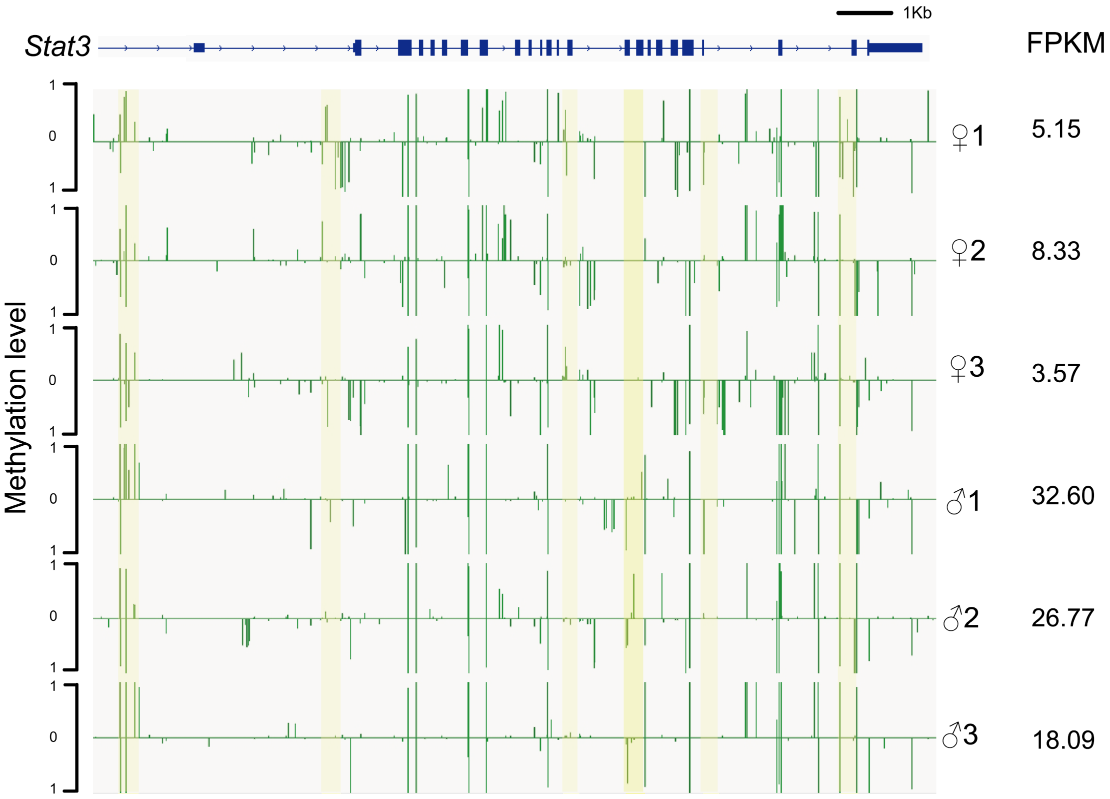

Figure S6. DNA methylation profiles of *Stat3*.

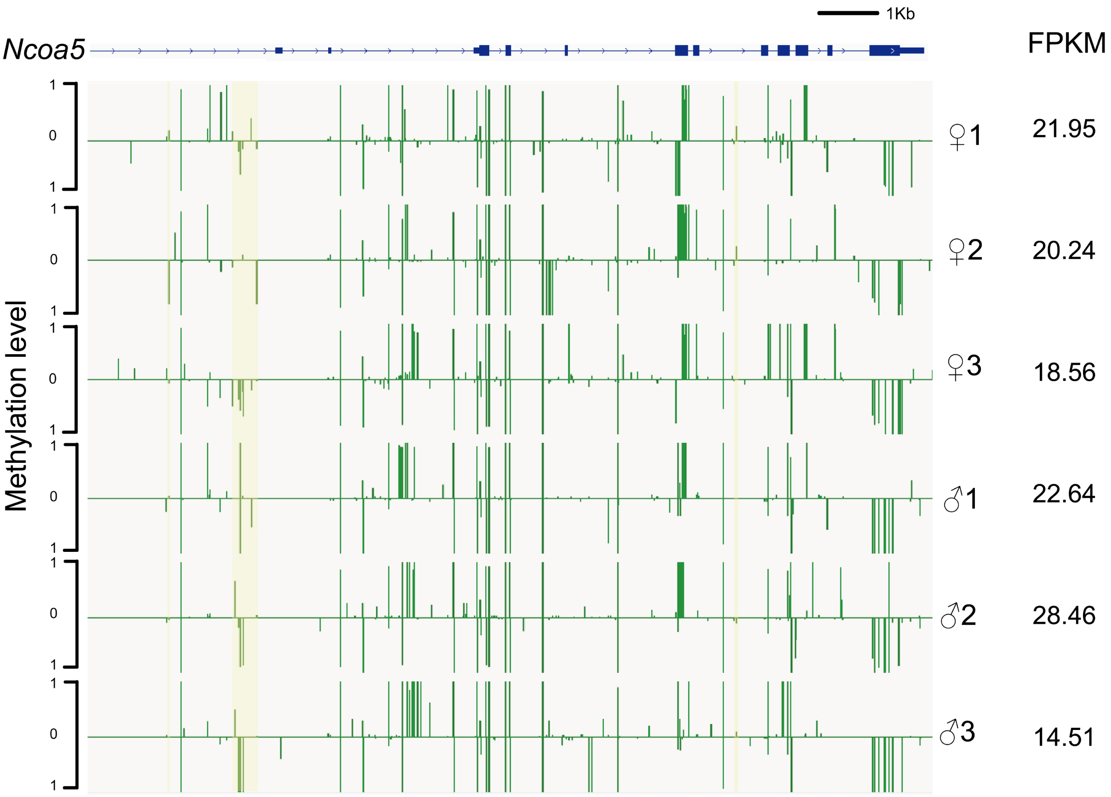

Figure S7. DNA methylation profiles of *Ncoa5*.

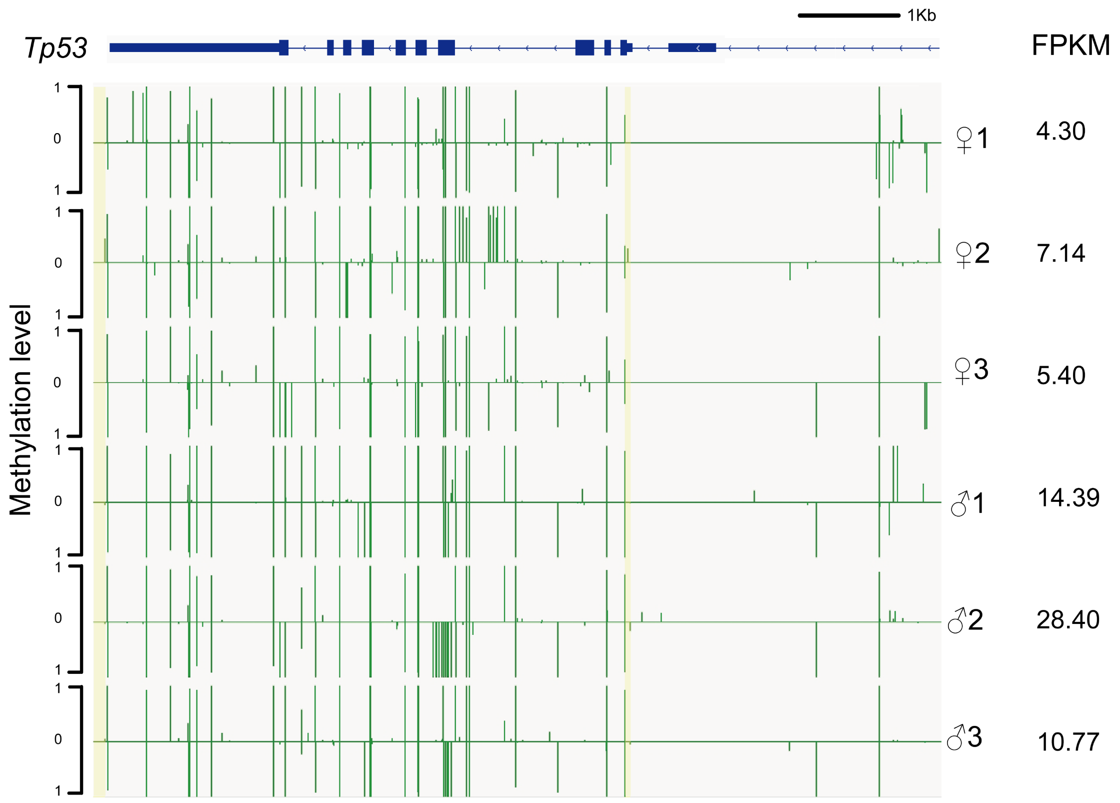

Figure S8. DNA methylation profiles of *Tp53*.

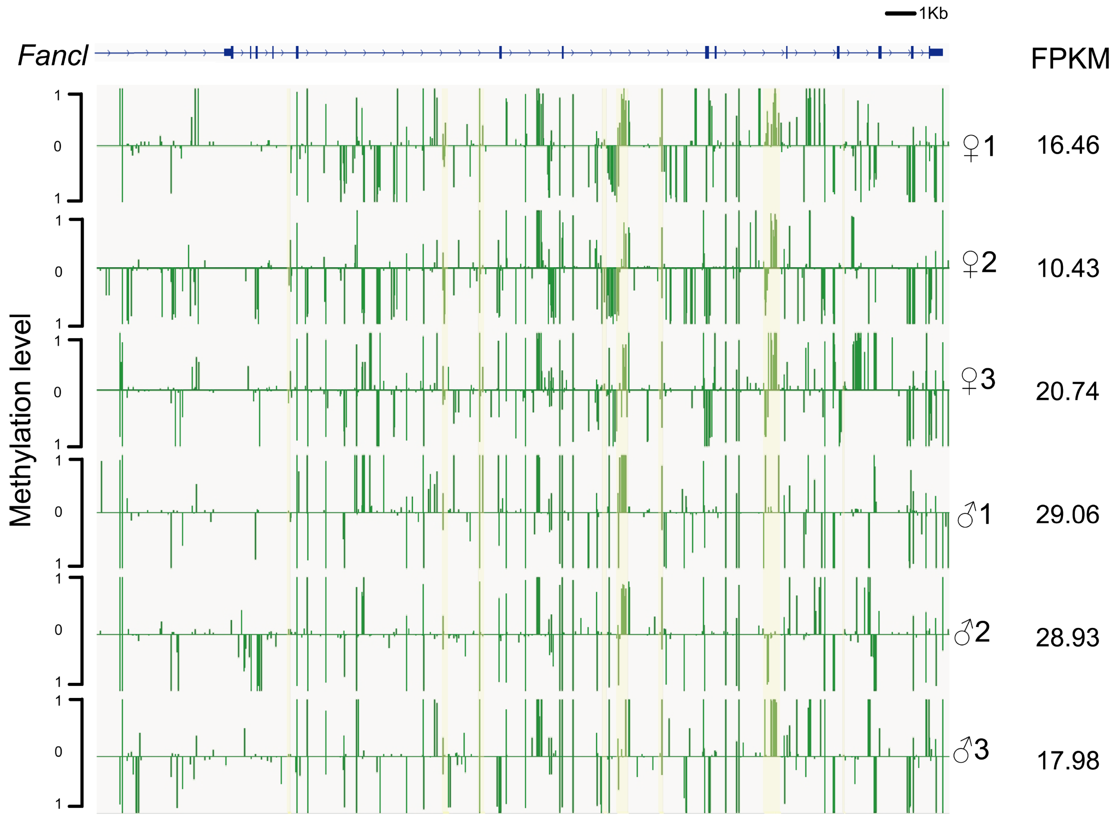

Figure S9. DNA methylation profiles of *Fancl*.

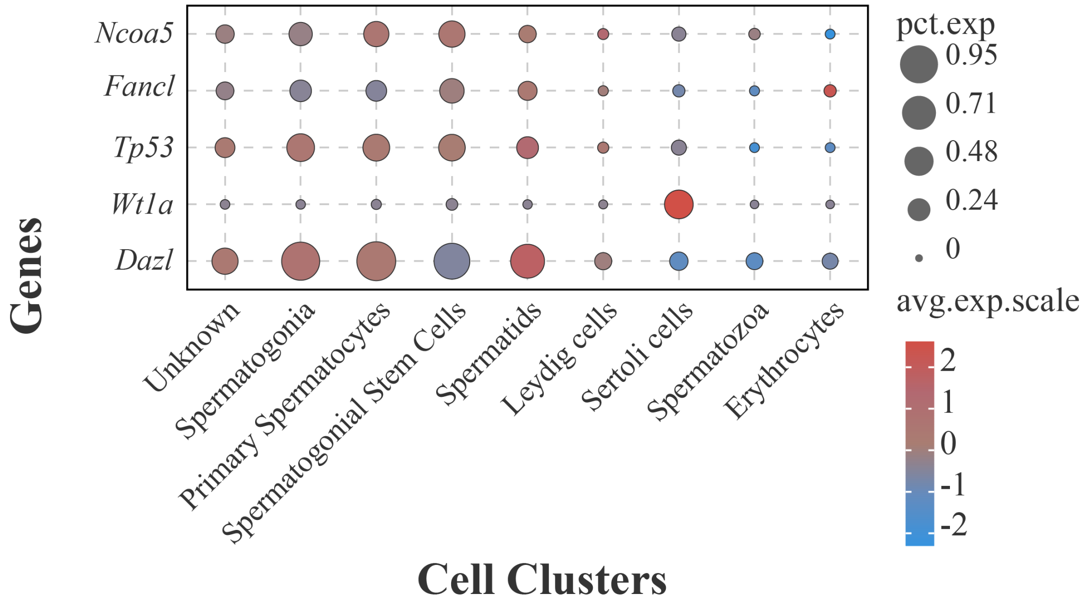

Figure S10. Cell-type-specific expression profiles of five selected differential alternative splicing genes in the adult testis of *S. argus*. Dot plot illustrating the expression distribution of *Ncoa5*, *Fancl*, *Tp53*, *Wt1a*, and *Dazl* across various testicular cell clusters based on scRNA-seq data. The size of each dot (pct.exp) indicates the proportion of cells within a specific cluster that express the target gene. The color gradient (avg.exp.scale) represents the average scaled expression level, ranging from blue (low expression) to red (high expression). Note the distinct compartmentalization: *Dazl*, *Tp53*, and *Fancl* are predominantly enriched in germ-cell lineages (e.g., spermatogonia and primary spermatocytes), whereas *Wt1a* exhibits strict somatic specificity in Sertoli cells.
